# Association Between Circulating Gremlin 2 and β‐Cell Function Among Participants With Prediabetes and Type 2 Diabetes

**DOI:** 10.1111/1753-0407.70090

**Published:** 2025-04-24

**Authors:** Mengshan Ni, Yanru Chen, Weiqiong Gu, Yifei Zhang, Min Xu, Yanyun Gu, Yufei Chen, Yinmeng Zhu, Xiao Wang, Yaogan Luo, Yu Xu, Xu Lin, Yi Arial Zeng, Ruixin Liu, Jiqiu Wang

**Affiliations:** ^1^ Department of Endocrine and Metabolic Diseases Shanghai Institute of Endocrine and Metabolic Diseases, Ruijin Hospital, Shanghai Jiao Tong University School of Medicine Shanghai China; ^2^ Shanghai National Clinical Research Center for Metabolic Diseases, Key Laboratory for Endocrine and Metabolic Diseases of the National Health Commission of the PR China Shanghai National Center for Translational Medicine Shanghai China; ^3^ Shanghai Institute of Nutrition and Health, University of Chinese Academy of Sciences, Chinese Academy of Sciences Shanghai China; ^4^ Key Laboratory of Systems Biology Hangzhou Institute for Advanced Study, University of Chinese Academy of Sciences, Chinese Academy of Sciences Hangzhou China; ^5^ State Key Laboratory of Cell Biology, CAS Center for Excellence in Molecular Cell Science, Institute of Biochemistry and Cell Biology University of Chinese Academy of Sciences Shanghai China

**Keywords:** β‐cell function, calorie restriction, Gremlin 2, oral antidiabetic drug, prediabetes, type 2 diabetes

## Abstract

**Aim:**

Circulating Gremlin 2 (Grem2) has recently been linked to human obesity, but its role in type 2 diabetes (T2D) remains unclear. This study aims to explore the association of circulating Grem2 with β‐cell function.

**Methods:**

A post hoc analysis was conducted using data from three clinical trials, in which all participants underwent the oral glucose tolerance test (OGTT). Circulating Grem2 levels were measured at 0, 1, and 2 h during the OGTT. In Trial 1, Grem2 levels were compared between participants with T2D (*n* = 59) and without T2D (*n* = 119). We further examined changes in Grem2 levels in response to oral antidiabetic drugs in participants with T2D in Trial 2 (*n* = 67) and calorie restriction in participants with prediabetes in Trial 3 (*n* = 231). The relationship between Grem2 levels and β‐cell function was analyzed across all trials.

**Results:**

Fasting and 1‐h Grem2 levels were lower in participants with T2D compared with those without T2D (728 ± 25 vs. 649 ± 31 pg/mL, *p* = 0.020; 631 ± 26 vs. 537 ± 31 pg/mL, *p* = 0.007). Fasting Grem2 levels were restored after antidiabetic treatment (550 ± 12 vs. 575 ± 12 pg/mL, *p* = 0.019), and 1‐h Grem2 levels increased following calorie restriction (1118 ± 89 vs. 1144 ± 90 vs. 1253 ± 89 pg/mL, *p* for trend = 0.002). The 1‐h Grem2 levels were positively associated with β‐cell function assessed by the oral disposition index and HOMA‐β.

**Conclusion:**

Reduced circulating Grem2 levels are associated with impaired β‐cell function in T2D, and could be restored through antidiabetic interventions.

**Trial Registration:** ClinicalTrials.gov: NCT01959984, NCT01758471, NCT03856762

## Graphical abstract

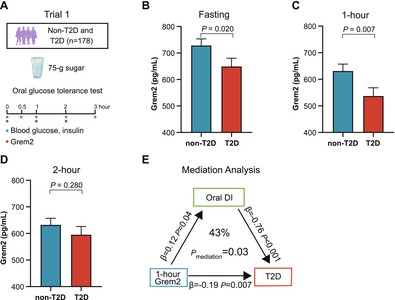

**Abbreviations**
AUC: area under the curve
BMP: bone morphogenetic protein
CI: confidence interval
DI: disposition index
GLP‐1: glucagon‐like peptide‐1
Grem2: Gremlin 2
OGTT: oral glucose tolerance test
T2D: type 2 diabetes

**Summary**

- Circulating Grem2 levels were significantly lower in participants with T2D compared with non‐T2D individuals and were restored by antidiabetic treatment and increased following calorie restriction.
- Circulating 1‐h Grem2 levels during the OGTT were positively associated with β‐cell function measured by the oral disposition index and HOMA‐β.
- Circulating Grem2 may serve as a potential biomarker and therapeutic target for diabetes.

## 1 Introduction

Type 2 diabetes (T2D) is a heterogeneous endocrine and metabolic disorder characterized by varying degrees of insulin resistance and/or dysfunction of pancreatic β‐cells [1]. T2D emerges when pancreatic β‐cells are unable to secrete sufficient insulin to overcome peripheral insulin resistance [2, 3]. Preservation of β‐cell secretory function is therefore critical to maintaining glucose homeostasis and preventing the onset of diabetes in at‐risk populations.

Insulin secretion from pancreatic β‐cells is a tightly regulated process, tuned by the precise coordination of various extracellular signals, including nutrients and hormones [4]. Currently, hormones and hormone analogs have emerged as compelling drug targets for T2D [5, 6]. Notably, therapies targeting glucagon‐like peptide‐1 (GLP‐1), a well‐established incretin hormone to enhance nutrient‐induced insulin release, have broadened treatment options and facilitated individualized management strategies for T2D [7, 8]. In particular, GLP‐1 receptor agonists (GLP‐1RAs) have garnered considerable attention due to additional benefits like weight loss and cardiovascular protection [9, 10, 11]. Nevertheless, persistent unmet patient needs, such as minimal adverse effects, longer lasting metabolic effects, and preventing a continuing decline in β‐cell function [5, 12], necessitate the discovery and development of new targets. Since the discovery of GLP‐1 in the early 1980s, there has been limited progress in identifying other hormones directly amplifying insulin secretion. Hence, it is imperative to identify novel circulating proteins potentially involved in diabetes pathophysiology, particularly those controlling insulin secretion.

Gremlin 2 (Grem2), an endogenous antagonist of bone morphogenetic proteins (BMPs), belongs to the DAN family [13]. Certain DAN family members, especially Gremlin 1, have been implicated in obesity, T2D, and metabolic dysfunction‐associated steatotic liver disease/metabolic dysfunction‐associated steatohepatitis (MASLD/MASH) [14, 15]. Several mouse studies suggest that Grem2 in kidney cells may play a role in diabetic nephropathy, a complication of T2D [16, 17, 18]. Additionally, the antidiabetic medication empagliflozin, an SGLT2 inhibitor, has been found to target hepatic Grem2 to alleviate metabolic dysfunction‐associated fibrosis in a MASLD mouse model [19]. However, little is known about the role of circulating Grem2 in metabolic diseases in humans. In a recent study, our team first reported circulating Grem2 in association with human central obesity [20]. Given obesity is tightly linked with diabetes and β‐cell function [21, 22], we speculated whether circulating Grem2 is associated with the development and treatment of diabetes in humans. However, to our knowledge, no study has yet explored the relationship.

Thus, we included three independent clinical studies to determine whether circulating Grem2 levels are altered in T2D and after therapeutic interventions, as well as their association with β‐cell function. Through this comprehensive study design, we will provide evidence of the impact of diabetes status and therapeutic interventions on circulating Grem2 levels and their relationship with β‐cell function.

## 2 Methods

### 2.1 Study Design and Participants

We performed a post hoc analysis using the dataset and blood sample measurements from three of our independent clinical trials [23, 24, 25]. In each trial, participants underwent the oral glucose tolerance test (OGTT), allowing for the evaluation of β‐cell function and measurement of circulating Grem2 levels at both fasting and post‐glucose load. Details of the design, setting, participants, and data collection of these studies have been reported previously, and characteristics of participants are summarized in Tables S1–S3. The study protocols were all approved by the Institutional Review Board of Ruijin Hospital, Shanghai Jiao Tong University School of Medicine, and conducted in accordance with the principles of the Helsinki Declaration. Informed consent was obtained from each participant.

**FIGURE 1:**
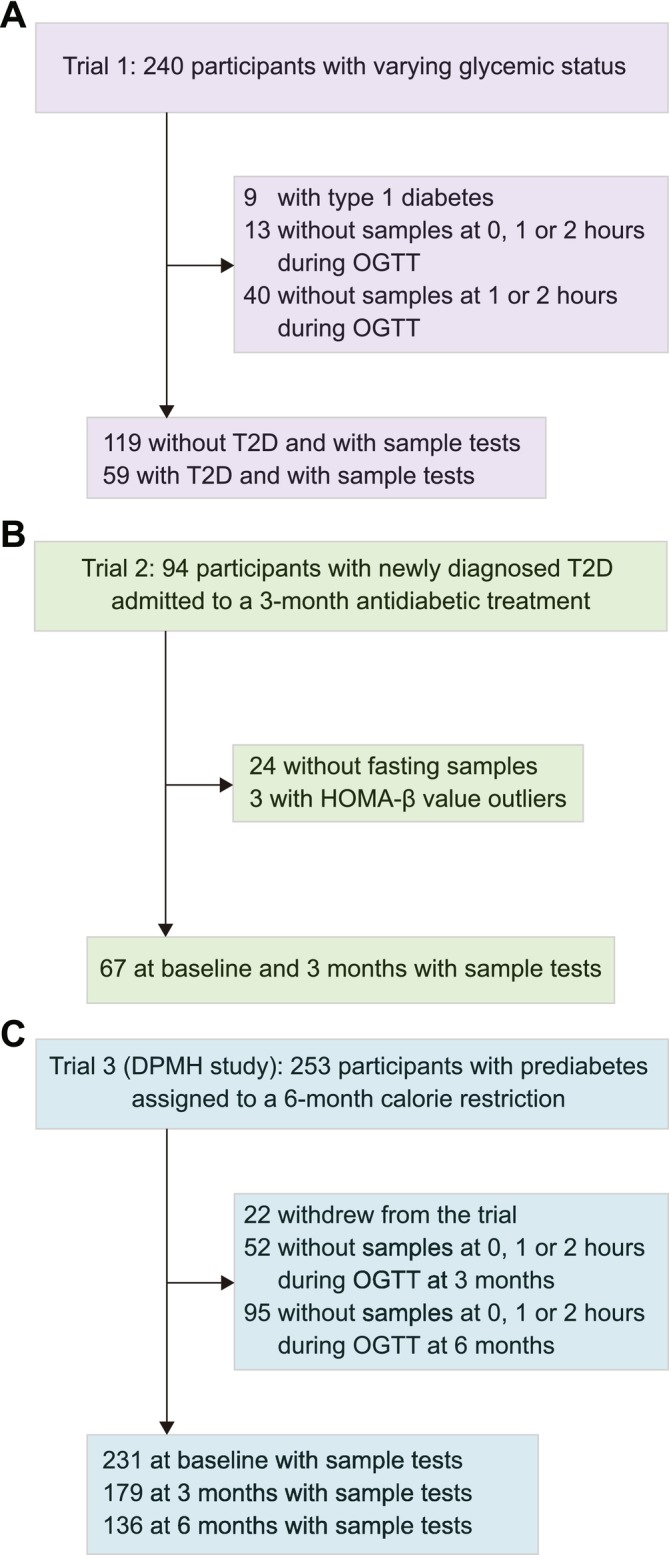
Flowchart of the study population in Trial 1 (A), Trial 2 (B), and Trial 3 (C).

All participants in the original study underwent the OGTT, during which both glucose and insulin levels were measured to assess β‐cell function indices. Only participants with available blood samples for Grem2 measurement were included in the present study. The study population flowchart is shown in Figure 1. Briefly, the first trial was a two‐way crossover randomized trial initially designed to compare the profiles of postprandial glucose, insulin, glucagon, and incretin levels following the consumption of either 75‐g oral glucose (a monosaccharide) or 100‐g standard noodles (a polysaccharide) in participants with T2D, T1D, or without diabetes (ClinicalTrials.gov) [23]. In the present analysis, 178 participants who underwent the 3‐h, 75‐g OGTT were included after excluding those diagnosed with type 1 diabetes or unavailable blood samples for Grem2 measurement. The second two‐arm randomized trial recruited participants with newly diagnosed T2D who were treated with either Acarbose or Glipizide for 3 months (ClinicalTrials.gov) [24]. In the present analysis, 67 participants were included after excluding those whose blood samples at baseline and 3 months were not available for Grem2 measurement or HOMA‐β outliers. The third trial, known as the Dietary Pattern and Metabolic Health (DMPH) trial, was a three‐arm randomized controlled trial comparing the effects of three isocaloric‐restricted diets (~25% calorie restriction) on body weight and glucose homeostasis in participants with prediabetes (fasting glucose ≥ 5.6 mmol/L) (ClinicalTrials.gov) [25]. Participants were randomly assigned to a Mediterranean diet, Jiangnan diet (a habitual diet originating from Southeast China), or control diet, and underwent a 5‐weekday full‐feeding regimen for 6 months. In the present analysis, a total of 231 participants at baseline were included after excluding those who left the trial after less than 3 months; 179 participants at 3 months and 136 participants at 6 months were included after excluding those who did not complete a 2‐h, 75‐g OGTT.

### 2.2 Grem2 Measurement

Blood samples were collected and stored at −80°C until use. Circulating Grem2 levels were determined using a commercial Grem2 ELISA kit (R&D systems, Minneapolis, USA) according to the manufacturer's protocol. The assay owes high specificity and sensitivity to human Grem2, as described previously [20].

### 2.3 Assessment of β‐Cell Function

Insulin secretion is coupled to insulin sensitivity through a hyperbolic relationship; hence, insulin secretion is expressed relative to insulin sensitivity (i.e., the disposition index [DI]) to accurately assess β‐cell function [26]. The oral DI calculated from the OGTT was used as a main indicator to evaluate β‐cell function [27]. The HOMA‐β was also calculated as a secondary measure of β‐cell function [28]. The oral DI, also known as insulin secretion‐sensitivity index‐2 (ISSI‐2), was calculated by multiplying measures of insulin secretion (the ratio of the area under the insulin curve to the area under the glucose curve) and insulin sensitivity (Matsuda index) [29, 30]. The trapezoidal method determined the area under the curve (AUC) for blood glucose and insulin. Insulin sensitivity was estimated by using the Matsuda index = 10 000/√[(fasting insulin (mU/L) × fasting glucose (mg/dL)) × (mean OGTT insulin (mU/L)) × (mean OGTT glucose (mg/dL))].

### 2.4 Statistical Analysis

Continuous variables are presented as means ± SEM, and categorical variables are presented as numbers (%). Data were analyzed using R Studio version 2022.12.0 and R version 4.2.2. A two‐tailed *p* value of less than 0.05 was considered statistically significant.

Given the relationship between Grem2 and obesity, body mass index (BMI) was included as a potential confounder in all analysis models. Multivariable linear regression was used to determine differences in Grem2 levels between participants without and with T2D, adjusting for age, sex, BMI, lipid‐lowering drug use, and antihypertensive drug use. For repeated measures, linear mixed‐effects models were used to investigate differences in Grem2 levels between different time points (hours during OGTT or months during the interventional trial), adjusting for age, sex, BMI, lipid‐lowering drug use, antihypertensive drug use, and other variables indicated in corresponding models. All post hoc analyses were performed with R package “emmeans” version 1.8.6 using Bonferroni correction for multiple comparison. The association between Grem2 and oral DI and HOMA‐β was analyzed with multivariable linear regression model, adjusted for suspected covariates: age, sex, BMI, lipid‐lowering drug use, and antihypertensive drug use. To ensure comparability of the effect sizes, coefficient estimates were standardized (the variables were centered and then divided by the SD so that the transformed variable had an expectancy value of 0 and a statistical variance of 1) and presented with 95% confidence interval (CI). Causal mediation analysis was performed by using the R package “mediation” version 4.5.0. Unstandardized indirect effects were computed for each of 10 000 bootstrapped samples, and the 95% CI was computed by determining the indirect effects at the 2.5th and 97.5th percentiles.

## 3 Results

### 3.1 Circulating Grem2 Levels Decline in Participants With T2D

**FIGURE 2:**
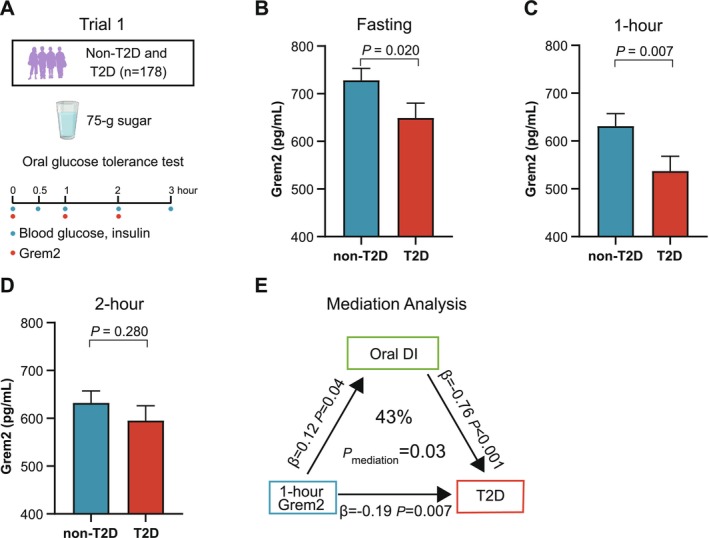
Grem2 levels decline in participants with type 2 diabetes. (A) Trial 1 comprising participants (*n* = 178) without type 2 diabetes (*n* = 119) and with type 2 diabetes (*n* = 59) who underwent a 3‐h, 75‐g oral glucose tolerance test. (B–D) Circulating Grem2 levels at 0 (B), 1 (C), and 2 h (D) in participants without and with type 2 diabetes. Data are expressed as estimated marginal mean ± SEM. Statistical significance was determined by multivariable linear regression models, adjusted for age, sex, BMI, lipid‐lowering drug use, and antihypertensive drug use. (E) Results of mediation analysis, demonstrating that oral disposition index mediated 43% of the effects of 1‐h Grem2 on type 2 diabetes. The *β*‐coefficients and *p* values are provided, along with the percentage of causal mediation effect. DI, disposition index; T2D, type 2 diabetes.

We first evaluated circulating Grem2 levels in 178 participants (59 with T2D and 119 without diabetes) who underwent a 3‐h OGTT (Figure 2A). The basic characteristics of the subtype participants are summarized in Table S1. Circulating Grem2 levels were measured at 0, 1, and 2 h during the OGTT. The levels significantly decreased at 1 h after glucose loading compared with baseline (*p* < 0.001), with no significant difference observed between the 1‐ and 2‐h time points (*p* = 1.00) (Figure S1A). Fasting Grem2 levels were lower in participants with T2D compared with those without T2D (728 ± 25 vs. 649 ± 31 pg/mL, *p* = 0.020) (Figure 2B), with an even more pronounced reduction observed at 1 h (631 ± 26 vs. 537 ± 31 pg/mL, *p* = 0.007) (Figure 2C). However, no significant difference in Grem2 levels was detected at the 2‐h time point (632 ± 25 vs. 595 ± 31 pg/mL, *p* = 0.280) (Figure 2D).

The associations between Grem2 levels at different OGTT time points and oral DI and HOMA‐β, both indicative of β‐cell function, were analyzed by multivariable linear regression with adjustments for age, sex, BMI, lipid‐lowering drug use, and antihypertensive drug use. The 1‐h Grem2 levels were positively associated with both oral DI (*β* = 0.12, *p* = 0.039) and HOMA‐β (*β* = 0.15, *p* = 0.030) (Table 1).

**TABLE 1:** Associations between circulating Grem2 levels and β‐cell function assessed by OGTT (*n* = 178).

Grem2 levels during OGTT (pg/mL)	Estimates for oral disposition index		Estimates for HOMA‐β	
	*β* (95% CI)	*p*	*β* (95% CI)	*p*
Fasting Grem2	0.06 (−0.09, 0.19)	0.403	0.00 (−0.13, 0.13)	0.989
1‐h Grem2	0.12 (0.01, 0.24)*	**0.039**	0.15 (0.01, 0.28)*	**0.030**
2‐h Grem2	0.03 (−0.10, 0.17)	0.640	0.03 (−0.10, 0.16)	0.677

*Note:* Associations between circulating Grem2 levels at different timepoints and oral disposition index, HOMA‐β were analyzed using multivariable linear regression adjusted for age, sex, BMI, lipid‐lowering drug use, and antihypertensive drug use. The standardized *β*‐coefficient and 95% CI, and *p* value obtained from multivariable linear regression were presented. Bold values indicate statistically significant results (*p* < 0.05). *p < 0.05, **p < 0.01.

Abbreviations: HOMA‐β, homeostasis model assessment of β‐cell function; OGTT, oral glucose tolerance test.

We further used oral DI as a potential mediation factor between Grem2 and T2D in the causal mediation analysis. The results revealed that the oral DI accounted for 43% of the effects of 1‐h Grem2 levels on T2D (*p*_mediation_ = 0.028) (Figure 2E).

### 3.2 Circulating Grem2 Levels Are Restored by Oral Antidiabetic Drug in Participants With Treatment‐Naïve T2D

**FIGURE 3:**
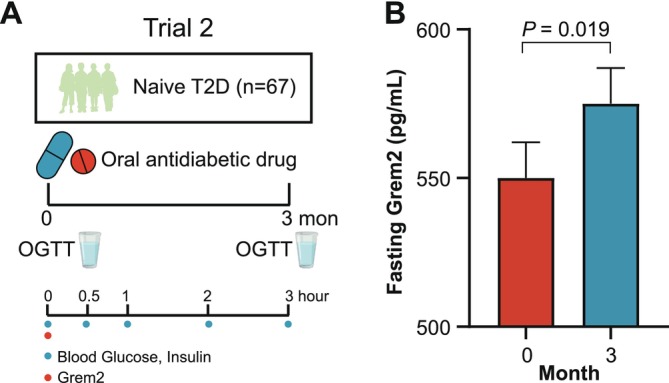
Grem2 levels are restored by antidiabetic treatment in participants with treatment‐naïve type 2 diabetes. (A) Trial 2 comprising 67 participants with treatment‐naïve type 2 diabetes who underwent a 3‐month treatment with the oral antidiabetic drug. (B) Elevation of fasting Grem2 levels after antidiabetic treatment. Data are expressed as estimated marginal mean ± SEM. Statistical significance was determined by linear mixed‐effects model, adjusted for age, sex, drug types, changes of BMI, lipid‐lowering drug use, and antihypertensive drug use, and baseline values.

Given the association of Grem2 with β‐cell function, we next sought to validate these findings in interventional trials that ameliorated β‐cell dysfunction in participants with T2D. The second trial was a randomized, two‐arm clinical trial in which 67 participants with treatment‐naïve T2D were treated with oral antidiabetic drugs, either Glipizide or Acarbose for 3 months (Figure 3A). Despite the availability of only fasting samples for Grem2 measurement, a significant elevation in fasting Grem2 levels was detected at the end of 3‐month antidiabetic treatment (550 ± 12 vs. 575 ± 12 pg/mL, *p* = 0.019) (Figure 3B), alongside enhancements in both oral DI and HOMA‐β among the participants (Table S4).

### 3.3 Circulating Grem2 Levels Increase After Calorie Restriction in Participants With Prediabetes

**FIGURE 4:**
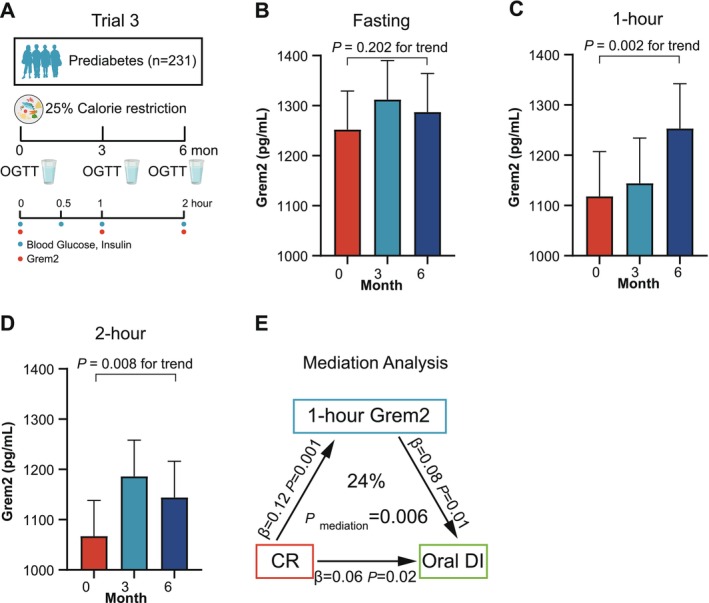
Grem2 levels increase after calorie restriction in participants with prediabetes. (A) Trial 3 comprising 231 participants with prediabetes who underwent a 6‐month 25% calorie restriction. (B–D) Circulating Grem2 levels at 0 (B), 1 (C), and 2 h (D) after calorie restriction. Data are expressed as estimated marginal mean ± SEM. Statistical significance was determined by linear mixed‐effects models, adjusted for age, sex, diet regimes, changes of BMI, lipid‐lowering drug use, antihypertensive drug use, and baseline values. (E) Results of mediation analysis, demonstrating that 1‐h Grem2 mediated 24% of the effects of caloric restriction on oral disposition index. The *β*‐coefficients and *p* values are provided, along with the percentage of causal mediation effect. CR, calorie restriction; DI, disposition index; T2D, type 2 diabetes.

Calorie restriction has also been established to improve β‐cell function [31, 32, 33, 34]. The third trial consisted of 231 participants with prediabetes undergoing a 25% calorie restriction for 6 months (Figure 4A). These participants underwent three OGTT tests at baseline, 3‐, and 6‐month after the intervention, respectively. Consistent with the findings in Trial 1, Grem2 levels before the calorie restriction intervention significantly decreased at 1 h after oral glucose loading (*p* < 0.001) but showed no further decline at the 2‐h time point (*p* = 1.00) (Figure S1B). As shown in Table S4, calorie restriction led to improved β‐cell function, as measured by oral DI, but not HOMA‐β in prediabetic participants. No significant change was observed in fasting Grem2 levels after calorie restriction (1252 ± 77 vs. 1312 ± 78 vs. 1287 ± 77 pg/mL, *p* = 0.202 for trend) (Figure 4B). However, calorie restriction resulted in an increase in 1‐h (1118 ± 89 vs. 1144 ± 90 vs. 1253 ± 89 pg/mL, *p* = 0.002 for trend) and 2‐h Grem2 levels (1067 ± 71 vs. 1186 ± 72 vs. 1144 ± 72 pg/mL, *p* = 0.008 for trend) (Figure 4C,D). Importantly, this increment of 1‐h Grem2 levels was positively associated with improved oral DI (*β* = 0.09, *p* = 0.006), after adjusting for age, sex, diet regimens, BMI changes, lipid‐lowering drug use, and antihypertensive drug use (Table 2).

**TABLE 2:** Associations between the changes of circulating Grem2 levels and the changes of β‐cell function assessed by OGTT (*n* = 231).

Grem2 levels during OGTT (pg/mL)	Estimates for oral disposition index		Estimates for HOMA‐β	
	*β* (95% CI)	*p*	*β* (95% CI)	*p*
Fasting Grem2	0.02 (−0.05, 0.09)	0.665	0.03 (−0.04, 0.10)	0.357
1‐h Grem2	0.09 (0.03, 0.15)**	**0.006**	−0.05 (−0.12, 0.01)	0.105
2‐h Grem2	0.05 (−0.02, 0.11)	0.170	−0.03 (−0.10, 0.04)	0.404

*Note:* Associations between the changes of circulating Grem2 levels at different timepoints and the changes of oral disposition index, HOMA‐β were analyzed using linear mixed‐effects models adjusted for age, sex, BMI, diets regimes, lipid‐lowering drug use, and antihypertensive drug use. The standardized *β*‐coefficient and 95% CI, and *p* value obtained from linear mixed‐effects models were presented. Bold values indicate statistically significant results (*p* < 0.05). *p < 0.05, **p < 0.01.

Abbreviations: HOMA‐β, homeostasis model assessment of β‐cell function; OGTT, oral glucose tolerance test.

Based on these clinical associations, it was assumed that the increase in 1‐h Grem2 mediates, at least in part, the improvements in β‐cell function induced by calorie restriction. The causal mediation analysis indicated that 1‐h Grem2 mediated 24% of the beneficial effect of calorie restriction on oral DI (*p*_mediation_ = 0.006) (Figure 4E).

## 4 Discussion

To our knowledge, this is the first study to investigate the circulating Grem2 levels in T2D and the association with β‐cell function across a spectrum of glycemia. Our findings demonstrated that circulating Grem2 levels decline in patients with T2D and rebound following various therapeutic interventions, including oral antidiabetic drugs and calorie restriction. Importantly, we identified a positive association between 1‐h Grem2 levels and β‐cell function assessed by OGTT. Collectively, our study provides novel insights into the role of Grem2, a newly identified circulating protein, in T2D and β‐cell function.

We initially observed the magnitude of decrease in both fasting and 1‐h Grem2 levels among individuals with T2D. Given the impaired β‐cell function in T2D patients, we proposed that circulating Grem2 may contribute to β‐cell function in humans. This possibility was further supported by evidence showing that 1‐h Grem2 levels were significantly and positively correlated with key β‐cell function indices. Additionally, antidiabetic interventions, including oral antidiabetic drugs and calorie restriction, which are known to improve β‐cell function, also resulted in increased Grem2 levels. The rebound in 1‐h Grem2 levels following 6 months of calorie restriction remained positively associated with improvements in the oral DI, an integrated measure of β‐cell function indices derived from the glucose challenge. These findings prompt a reevaluation of Grem2's role in obesity and T2D, proposing that increased circulating Grem2 in obesity may act as a defensive response to counteract glucose homeostasis disturbances. Alternatively, the alterations in glucose homeostasis due to obesity may impair Grem2 signaling, resulting in a compensatory increase in Grem2 levels, akin to mechanisms seen in hyperinsulinemia and hyperleptinemia.

The ability to assess individual risk of impaired β‐cell function before the onset of T2D would enable the implementation of preventive lifestyle interventions or pharmacologic treatments. As the oral DI accurately represents β‐cell insulin secretory rates in the context of insulin resistance, it has been established as a metabolic predictor of progression to diabetes [35, 36]. The 1‐h Grem2 levels were positively associated with β‐cell function across the spectrum of glucose tolerance from normal to overt T2D, independent of age, sex, and adiposity. In consistency, for participants with prediabetes, even in the insulin‐compensated state [37, 38], higher 1‐h Grem2 levels were associated with better β‐cell function indicated by the oral DI independent of age, sex, and adiposity before calorie restriction (Figure S2). Accordingly, measuring 1‐h Grem2 in circulation could potentially be a relatively noninvasive and easily accessible method for assessing the risk of developing T2D. Of note, this does not diminish the importance of fasting Grem2 levels in clinical practice. Indeed, participants with T2D exhibited significantly lower fasting Grem2 levels, which could be significantly restored with antidiabetic treatment, indicating a possibility of the involvement of impaired fasting Grem2 in the pathogenesis of T2D. Nonetheless, the more pronounced decline of Grem2 and the more robust associations with DI specifically emerging in the post‐glucose state suggested that 1‐h Grem2 levels are more sensitive indicators of β‐cell function and may exert an impact in the early stage of T2D onset. Ultimately, the performance of Grem2 as a biomarker will need to be studied in larger cohorts.

The beneficial effects of calorie restriction on improving β‐cell function have been well‐documented by previous studies [31, 32, 33, 34], also supported by our research where prediabetic participants displayed increased oral DI following a 25% calorie restriction. However, the other mechanisms underlying the benefits of caloric restriction in β‐cell function are not fully understood, apart from weight loss. Intriguingly, although we previously showed that fasting Grem2 levels are linked with obesity, the relationship between circulating Grem2 and β‐cell function was independent of BMI. Participants who experienced weight loss following calorie restriction showed unchanged fasting Grem2 levels but increased post‐glucose circulating Grem2 levels. The increment in 1‐h Grem2 levels after a 6‐month intervention was positively associated with the improved oral DI, independent of weight loss. Notably, our causal mediation analysis further confirms the effect of 1‐h Grem2 levels on oral DI. Therefore, it could be speculated that the systemic augment of postprandial Grem2 might account for calorie restriction‐ induced recovery of β‐cell function beyond weight reduction. The mechanism by which calorie restriction triggers elevated Grem2 levels, however, remains an avenue for further exploration.

Another pertinent question is which organ(s) contribute to the circulating Grem2 pool. During OGTT, Grem2 levels were suppressed to some extent by blood glucose, different from the typical spike of gut‐derived incretin hormones such as GLP‐1. Although our previous work has suggested visceral adipose tissue contributes a small proportion (approximately 20%) of circulating Grem2 [20], classic adipokines such as leptin and adiponectin do not exhibit rapid changes after acute glucose stimulation [39]. Therefore, other organs may also contribute to circulating Grem2 levels, particularly given the fairly high expression of the *PRDC* gene (encoding Grem2 protein) in several organs such as the liver.

The main strength of our study lies in the use of multiple trials where all participants were screened by OGTT, allowing us to examine whether both fasting and post‐glucose Grem2 levels change following antidiabetic interventions and are associated with β‐cell function. However, our study has several limitations that should be acknowledged. Firstly, variations in Grem2 levels were observed across different trials, which may be attributable to factors such as differences in sample storage time and the availability of either serum or plasma for Grem2 measurement. Despite this, the methods for blood sample storage and measurement were consistent across three trials. Secondly, the findings of this study are based on participants of Chinese descent, and caution should be exercised when generalizing these results to other populations due to reported differences in insulin sensitivity and insulin secretion among various ethnic groups [40]. Additionally, due to the post hoc nature of this study and the relatively small sample size, larger prospective studies are needed to validate our findings. A final limitation is that the current data cannot establish a direct link between β‐cells and Grem2. However, we have observed that recombinant Grem2 protein enhances glucose‐stimulated insulin secretion in freshly isolated mouse islets (data not shown). Our team is actively investigating the underlying mechanisms and anticipates obtaining more comprehensive results in the near future.

In conclusion, despite currently limited knowledge of the biological function of Grem2 in diabetes, our identification of circulating Grem2 in response to antidiabetic interventions and its consistent association with β‐cell function across a spectrum of glycemia in three independent trials opens new possibilities for diabetes prevention and therapy.

## Author Contributions

J.W., R.L., and M.N. conceived and designed this study. Y.Z. led the original trial studying different carbohydrate tolerance tests. Y.G. led the original oral antidiabetic drug trial. X.L. and J.W. led the original DPMH trial. M.N. and M.X. were involved in designing the analysis methods. M.N. conducted the analyses and wrote the first draft of this manuscript. All authors contributed to data acquisition, interpreted the results, edited, reviewed, and approved the final version of the manuscript. J.W. and R.L. are the guarantors of this work and, as such, had full access to all the data in the study and take responsibility for the integrity of the data and the accuracy of the data analysis.

## Conflicts of Interest

The authors declare no conflicts of interest.

## Supporting information


**Table S1.** Characteristics of participants in Trial 1.
**Table S2.** Characteristics of participants before and after 3‐month antidiabetic treatment in Trial 2.
**Table S3.** Characteristics of participants at baseline, 3‐, and 6‐month calorie restriction in Trial 3.
**Table S4.** β‐cell function indices and circulating Grem2 levels in participants across three trials.
**Figure S1.** Rapid decline of Grem2 levels upon oral glucose load.
**Figure S2.** Increment of oral disposition index according to tertiles of Grem2 at 1 h at baseline in Trial 3.

## Data Availability

The datasets used and/or analyzed during the current study are available from the corresponding author on reasonable request.
