# Supplementary material for: Association Between Circulating Gremlin 2 and β‐Cell Function Among Participants With Prediabetes and Type 2 Diabetes
Source: J Diabetes. 2025 Apr 24;17(4):e70090. doi: 10.1111/1753-0407.70090 (PMC12018890; doi:10.1111/1753-0407.70090)
Supplement: Supplementary file 1 — Table S1. Characteristics of participants in Trial 1. Table S2. Characteristics of participants before and after 3‐month antidiabetic treatment in Trial 2. Table S3. Characteristics of participants at baseline, 3‐, and 6‐month calorie restriction in Trial 3. Table S4. β‐cell function indices and circulating Grem2 levels in participants across three trials. Figure S1. Rapid decline of Grem2 levels upon oral glucose load. Figure S2. Increment of oral disposition index according to tertiles of Grem2 at 1 h at baseline in Trial 3. [file JDB-17-e70090-s001.docx]

**Supplementary information**

**Supplementary Tables 1-4**

**Supplementary Table 1. Characteristics of participants in Trial 1**

| **Variable** | **Non-diabetes (n=119)** | **Diabetes (n=59)** | ***P* value** |
| --- | --- | --- | --- |
| Age, yr | 42.89 (14.09) | 51.42 (10.26) | <0.001 |
| Male sex, no. (%) | 55 (46%) | 39 (66%) | 0.012 |
| BMI, kg/m^2^ | 24.82 (4.00) | 25.70 (3.51) | 0.151 |
| HbA1c, % | 5.34 (0.42) | 7.50 (1.57) | <0.001 |
| Diabetes duration, mth | 0 (0) | 52.88 (67.88) | <0.001 |
| **Glucose** |  |  |  |
| Fasting, mmol/L | 5.33 (0.56) | 8.45 (2.98) | <0.001 |
| 2-hour, mmol/L | 7.07 (1.73) | 16.55 (4.47) | <0.001 |
| AUC, mmol*min/L | 1327.98 (258.66) | 2667.30 (697.77) | <0.001 |
| **Insulin** |  |  |  |
| Fasting, µIU/mL | 10.63 (7.55) | 11.61 (7.47) | 0.412 |
| 2-hour, µIU/mL | 82.30 (93.75) | 61.99 (56.95) | 0.128 |
| AUC, µIU*min/mL | 13528.31 (12435.19) | 8115.69 (6395.00) | <0.001 |

Data are n (%) or mean (SD). Differences between non-diabetic and diabetic subjects were evaluated using the Student’s t test for continuous variables, and the χ^2^ test for categorical variables.

Abbreviations: AUC, the area under the curve.

**Supplementary Table 2. Characteristics of participants before and after 3-month antidiabetic treatment in Trial 2**

| **Variable** | **Pre-treatment**  **(n=67)** | **Post-treatment**  **(n=67)** | ***P* value** |
| --- | --- | --- | --- |
| Age, yr | 53.20 (6.92) |  |  |
| Male sex, no. (%) | 47 (66) |  |  |
| BMI, kg/m^2^ | 25.97 (3.28) | 25.73 (3.30) | 0.438 |
| HbA1c, % | 7.59 (0.87) | 6.39 (0.58) | <0.001 |
| **Glucose** |  |  |  |
| Fasting, mmol/L | 7.74 (1.45) | 6.75 (1.04) | <0.001 |
| 2-hour, mmol/L | 14.61 (2.80) | 10.14 (2.56) | <0.001 |
| AUC, mmol*min/L | 2311.58 (372.96) | 1692.18 (346.38) | <0.001 |
| **Insulin** |  |  |  |
| Fasting, µIU/mL | 10.39 (7.73) | 11.68 (15.28) | 0.116 |
| 2-hour, µIU/mL | 49.63 (28.21) | 42.87 (26.77) | 0.224 |
| AUC, µIU*min/mL | 6566.06 (3203.37) | 5805.04 (3317.20) | 0.118 |

Data are n (%) or mean (SD).

Abbreviations: AUC, the area under the curve.

**Supplementary Table 3. Characteristics of participants at baseline, 3-month, and 6-month calorie restriction in Trial 3**

| **Variable** | **Baseline**  **(n=231)** | **3-month**  **(n=179)** | **6-month**  **(n=136)** | ***P* value** |
| --- | --- | --- | --- | --- |
| Age, yr | 37.84 (8.70) | 38.69 (8.92) | 39.10 (8.98) |  |
| Male sex, no. (%) | 196 (85) | 150 (84) | 118 (87) |  |
| BMI, kg/m^2^ | 26.71 (3.00) | 25.17 (2.83) | 24.66 (2.65) | <0.001 |
| HbA1c, % | 5.85 (0.59) | 5.92 (0.53) | 6.01 (0.44) | 0.541 |
| **Glucose** |  |  |  |  |
| Fasting, mmol/L | 6.20 (0.72) | 6.09 (0.62) | 5.85 (0.48) | <0.001 |
| 2-hour, mmol/L | 7.91 (2.78) | 8.01 (2.41) | 7.37 (2.25) | <0.001 |
| AUC, mmol*min/L | 1066.66 (238.79) | 1120.86 (210.47) | 1030.44 (191.90) | 0.014 |
| **Insulin** |  |  |  |  |
| Fasting, µIU/mL | 15.73 (9.98) | 12.60 (10.44) | 11.18 (7.05) | <0.001 |
| 2-hour, µIU/mL | 105.83 (93.31) | 101.30 (94.54) | 88.53 (97.18) | <0.001 |
| AUC, µIU*min/mL | 12787.4 (9109.6) | 11753.8 (8128.7) | 11503.4 (10170.8) | <0.001 |

Data are n (%) or mean (SD).

Abbreviations: AUC, the area under the curve.

**Supplementary Table 4. β-cell function indices and circulating Grem2 levels in participants across three trials**

| Variable | Trial 1 | |  | *P* value |
| --- | --- | --- | --- | --- |
|  | Non-diabetes (n=119) | Diabetes (n=59) |  |  |
| Oral DI | 32.8 (0.9) | 9.5 (1.3) |  | **< 0.001***** |
| HOMA-β | 126.6 (8.4) | 67.0 (12.5) |  | **< 0.001***** |
| Fasting Grem2 | 728 (25) | 649 (31) |  | **0.020*** |
| 1-hour Grem2 | 631 (26) | 537 (31) |  | **0.007**** |
| 2-hour Grem2 | 632 (25) | 595 (31) |  | 0.280 |
|  | Trial 2 | |  |  |
|  | Baseline (n=67) | 3-month (n=67) |  |  |
| Oral DI | 9.3 (0.7) | 15.3 (0.7) |  | **< 0.001***** |
| HOMA-β | 49.2 (2.3) | 57.8 (2.3) |  | **0.008**** |
| Fasting Grem2 | 550 (12) | 575 (12) |  | **0.019*** |
|  | Trial 3 | | |  |
|  | Baseline (n=231) | 3-month (n=179) | 6-month (n=136) |  |
| Oral DI | 22.9 (0.4) | 22.85 (0.4) | 26.2 (0.5) | **< 0.001***** |
| HOMA-β | 114.8 (5.1) | 111.7 (5.4) | 111.9 (5.8) | 0.513 |
| Fasting Grem2 | 1252 (77) | 1312 (78) | 1287 (77) | 0.202 |
| 1-hour Grem2 | 1118 (89) | 1144 (90) | 1253 (89) | **0.002**** |
| 2-hour Grem2 | 1067 (71) | 1186 (72) | 1144 (72) | **0.008**** |

Data are presented as mean (SEM). Statistical significance was determined by multivariable linear regression models, adjusted for age, sex, BMI, lipid-lowering drugs, and antihypertensive drugs in Trial 1; linear mixed-effects model, adjusted for age, sex, drug types, changes of BMI, lipid-lowering drugs, antihypertensive drugs, and baseline values in Trial 2; and by linear mixed-effects models, adjusted for age, sex, diet regimes, changes of BMI, lipid-lowering drugs, antihypertensive drugs, and baseline values in Trial 3.

Abbreviations: DI, disposition index; HOMA-β, homeostasis model assessment for β-cell function.

**Supplementary Figures 1-2**

**
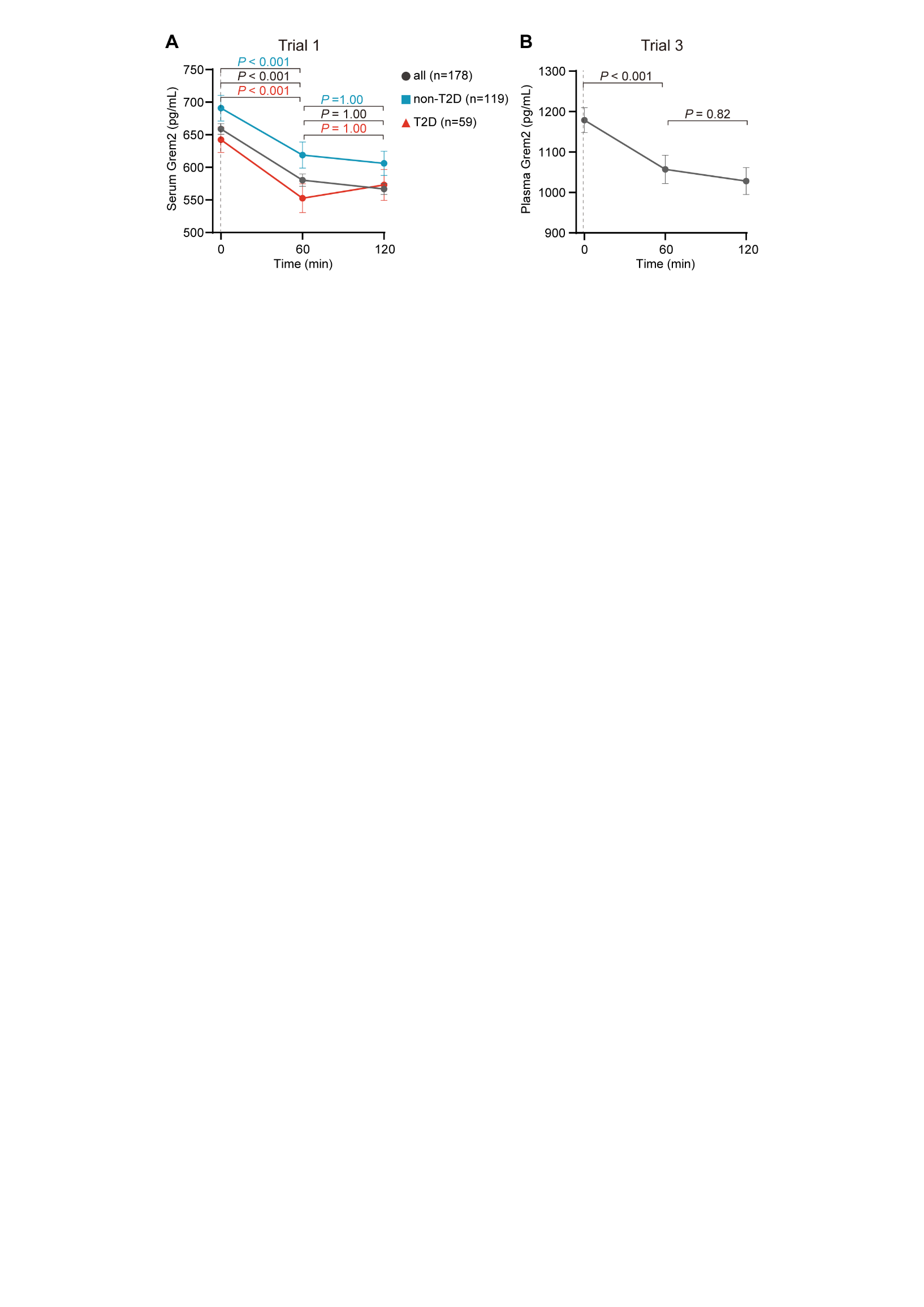
**

**Supplementary Figure 1. Rapid decline of Grem2 levels upon oral glucose load**

(A and B) Grem2 profiles during the oral glucose tolerance test in Trial 1 (A) and Trial 3 (B). Data are expressed as mean ± SEM. Statistical significance was determined by linear mixed-effects models, adjusted for age, sex, BMI, and baseline values, with Bonferroni correction for multiple comparisons.

**
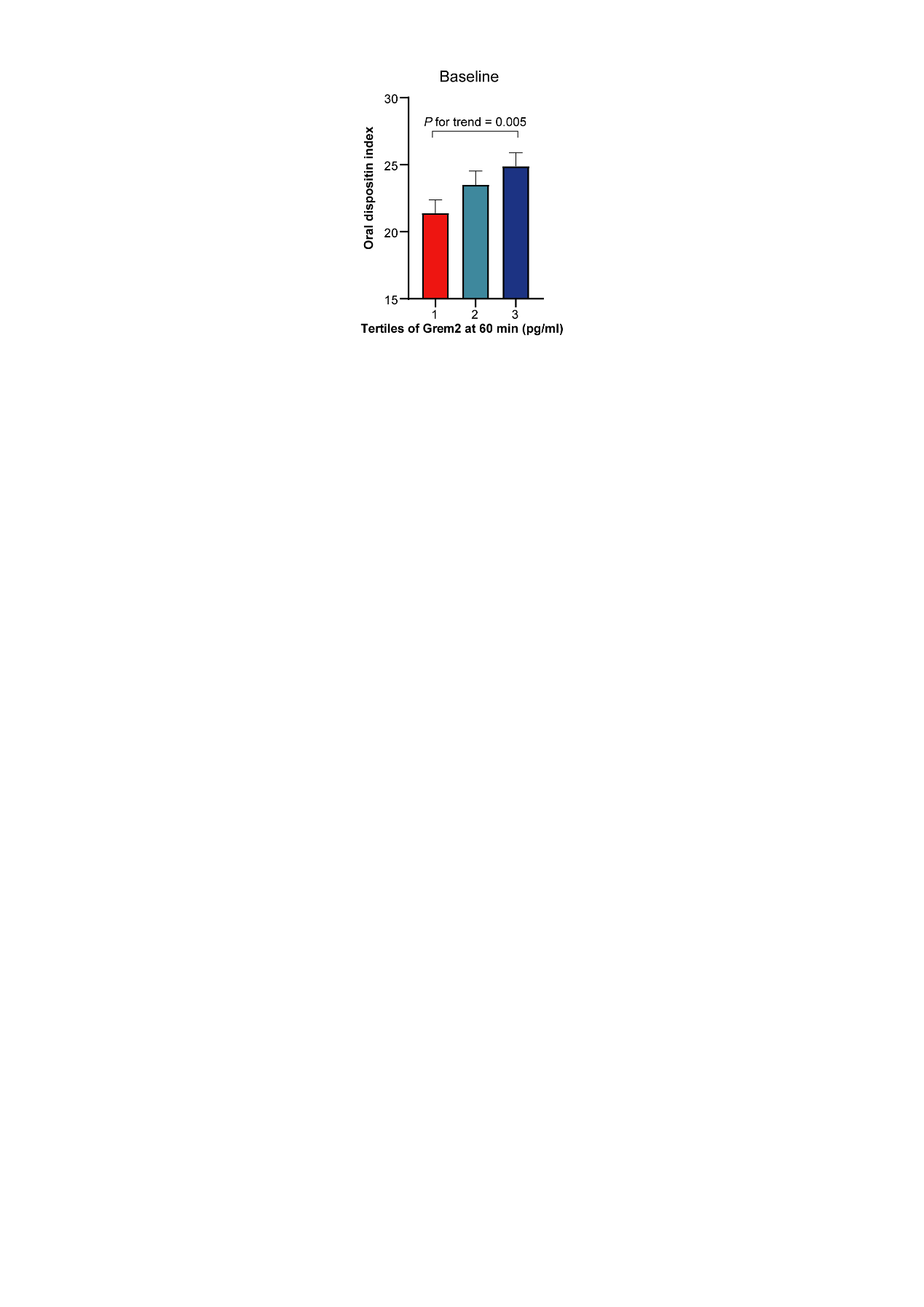
**

**Supplementary Figure 2. Increment of oral disposition index according to tertiles of Grem2 at 60min at baseline in Trial 3**

Data are expressed as means ±SEM. *P* for trend was determined by multivariable linear regression with tertiles and adjusted for age, sex and BMI.
